# Drug-Coated Balloons for Acute Myocardial Infarction: A Metaanalysis of Randomized Clinical Trials

**DOI:** 10.1155/2022/4018771

**Published:** 2022-12-27

**Authors:** Yuxuan Zhang, Delong Chen, Qichao Dong, Yi Xu, Jiacheng Fang, Huaqing Zhang, Jun Jiang

**Affiliations:** ^1^Department of Cardiology, Second Affiliated Hospital, College of Medicine, Zhejiang University, Hangzhou, China; ^2^Department of Cardiology, Ningbo First Hospital, Ningbo, China; ^3^Department of Clinical Engineering, Second Affiliated Hospital, College of Medicine, Zhejiang University, Hangzhou, China; ^4^Cardiovascular Key Laboratory of Zhejiang Province, Hangzhou, China

## Abstract

**Background:**

The role of a drug-coated balloon (DCB) in the treatment of acute myocardial infarction (AMI) is not well established.

**Methods:**

Five databases were searched for randomized controlled trials that compared DCB with stents in the treatment of AMI from their inception to 30 July 2021. The primary clinical endpoint was major adverse cardiac events (MACEs). Summary estimations were conducted using fixed-effects analysis complemented by several subgroups. The protocol was registered with PROSPERO (https://clinicaltrials.gov/ct2/show/CRD42021272886).

**Results:**

A total of 4 randomized controlled trials with 485 patients were included. On routine clinical follow-up, DCB was associated with no difference in the incidence of MACEs compared with control (risk ratio [RR] 0.59 [0.31 to 1.13]; *P*=0.11). DCB was associated with similar MACEs compared with drug-eluting stent and lower MACEs compared with bare-metal stent. There was no difference between DCB and control in terms of all-cause mortality, cardiovascular mortality, stent thrombosis, target lesion revascularization, and minimal lumen diameter during follow-up. However, DCB was associated with a lower incidence of myocardial infarction (RR 0.16 [0.03 to 0.90]; *P*=0.04) and lower late lumen loss (mean difference −0.20 [−0.27 to −0.13]; *P* < 0.00001).

**Conclusions:**

In treatment of patients with AMI, DCB might be a feasible interventional strategy versus control as it associated with comparable clinical outcomes. Future large-volume, well-designed randomized controlled trials to evaluating the role of the DCB in this setting are warranted.

## 1. Introduction

Acute myocardial infarction (AMI) with or without ST-segment elevation (STEMI or non-STEMI) is a common cardiac emergency with the potential for substantial morbidity and mortality [1]. Since the early 1990s, the management of acute myocardial infarction has improved significantly and is still evolving. Numerous studies have supported primary percutaneous coronary intervention (PPCI) with implantation of a permanent drug-eluting stent (DES) is the optimal strategy for the treatment of STEMI [2] and has been adopted by guidelines as class IA recommendation [3, 4]. For the treatment of non-STEMI, the results of RCTs and their metaanalysis highlight the role of risk stratification in the decision process and support a routine invasive strategy in high-risk patients [5–7]. However, stenting did not reduce the incidence of cardiac death or recurrent myocardial infarction (MI) [8]. In addition, implantation of permanent metal scaffolding led to an increased risk of late and very late stent thrombosis (ST), particularly in STEMI patients [9–11].

A drug-coated balloon (DCB) is a very attractive therapeutic alternative for percutaneous coronary intervention (PCI), which eliminates stent thrombosis, decreases patients' dependence on dual antiplatelet therapy, and reduces the rate of restenosis by leaving no metal behind [12]. A DCB-only strategy has already been shown to be safe and effective in the treatment of in-stent restenosis and small vessel disease [13, 14]. In patients with de novo coronary lesions, a large metaanalysis showed comparable safety and efficacy with the use of DCB regardless of the indication or comparator device [15]. Recently, several small-sized randomized trials have evaluated the feasibility of DCB for patients with AMI but not powered to assess the differences in clinical outcomes [16–19]. The effects of DCB in treatment of AMI are still less well known. We performed a metaanalysis to assess the clinical efficacy of DCB in the management of AMI.

## 2. Methods

This metaanalysis was performed pursuant to the Preferred Reporting Items for Systematic Reviews and Meta-Analysis (PRISMA) Guidelines [20] (Supplementary Table 1), and the protocol was registered with PROSPERO (https://clinicaltrials.gov/ct2/show/CRD42021272886).

### 2.1. Literature Search Strategy and Selection Criteria

Two investigators (Q.D. and D.C.) systematically and independently searched five databases, which including PubMed, Embase, Web of Science, the Cochrane Library, and ClinicalTrials.gov, from their inception to 30 July 2021. The following search terms, keywords, and controlled vocabularies were used: “coated balloon,” “eluting balloon,” “myocardial infarction,” “primary percutaneous coronary intervention,” “acute myocardial infarction,” and “randomized controlled trial” (Supplementary Table 2).

Eligible RCTs were supposed to meet the following inclusion criteria: (1) participants were adults with AMI intended for PPCI; (2) the interventions corresponded with the following candidate therapies: DCB implantation and DES implantation; (3) outcomes of endpoints were available; (4) studies beyond 6-monthfollow-up.

We excluded studies that met the following criteria: (1) nonrandomized trials; (2) trials used DCB plus predominantly bare-metal stent (BMS); (3) trials with a crossover design; (4) studies not published in English; (5) nonfull-text manuscript studies.

### 2.2. Data Extraction and Quality Assessment

Two investigators (Y.Z. and Y.X.) independently screened the titles, abstracts, and sequentially full articles. Then, they extracted data on the study design, baseline characteristics, and outcomes from full texts or published appendixes using prespecified forms. The primary clinical endpoint was major adverse cardiac events (MACEs, defined according to each study protocol). The secondary clinical endpoints included: target lesion revascularization (TLR); myocardial infarction (MI); cardiovascular mortality; all-cause mortality; and stent thrombosis. The following angiographic outcomes were assessed: minimum lumen diameter (MLD) and late lumen loss (LLL). Data extraction was under the instruction of the intention-to-treat principle. We appraised the quality of eligible studies according to the Cochrane Risk of Bias Tool [21]. In addition, a third investigator (J. J.) identified the accuracy of the information and handled the contradictions by consensus.

### 2.3. Statistical Analysis

Dichotomous outcomes and continuous outcomes were expressed as relative risk (RR) with 95% confidence intervals (CI) and weighted mean difference (WMD), respectively. Heterogeneity between trials was assessed using Cochran's test and means of *I*^2^ statistic [22]. Regardless of the heterogeneity of the included studies, random-effects statistical models were used for calculations of summary estimates and their 95% CI. Publication bias and sensitivity analysis were not assessed because of the small number of included articles. Subgroup analyses were aimed at exploring important clinical differences among that might be expected to alter the magnitude of treatment effect. *P* values of 0.05 were considered statistically significant. All analyses were performed using the Review Manager (version 5.4, The Nordic Cochrane Center, Købehvn, Denmark).

## 3. Results

### 3.1. Eligible Studies

**Figure 1:**
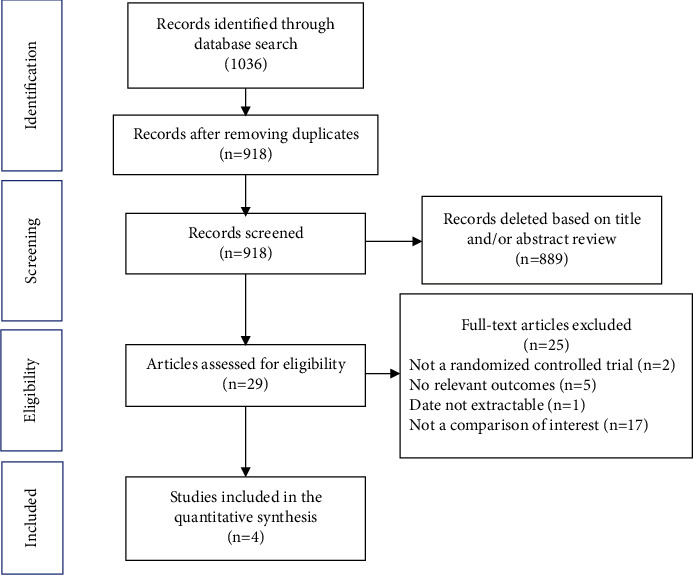
The flow diagram depicting the selection of studies included in the metaanalysis.

The systematic search identified 918 studies after removal of the duplicates. After assessment of the title and abstract, 29 studies were reviewed in full text for eligibility (Figure 1). A total of 4 randomized clinical trials were finally included in this metaanalysis, involving 485 patients (240 in the DCB group and 245 in the control group) [16–19]. Supplementary table 3 shows the baseline characteristics of participants. The SeQuent Please paclitaxel-coated balloon was used in two trials [16, 18], while other two trials used Yinyi (Liaoning) Biotech Bingo DCB [19] and Pantera Lux DCB [17], respectively. For the control group, the second-generation DESs were used in 3 trials [16, 17, 19]. In one trial, both second-generation DES and BMS were used, and a subgroup analysis was reported for the outcomes based on the stent type [18]. All trials were in low-risk category according to the Cochrane Risk of Bias Tool (Supplementary Figure 1).

### 3.2. Primary Endpoint

**Figure 2:**
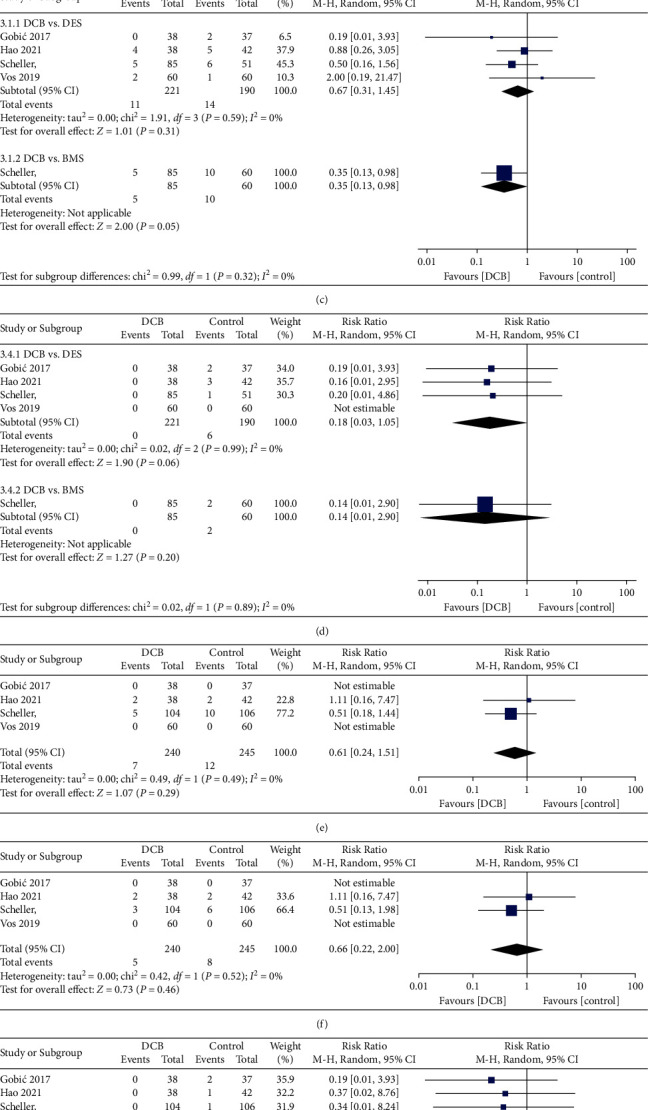
Summary plots for the clinical endpoint. Risk ratios of major adverse cardiac events (a), myocardial infarction (b), all-cause death (e), cardiovascular mortality (f), stent thrombosis (g), and target lesion revascularization (h); subgroup analysis for major adverse cardiac events (c) and myocardial infarction (d) according to indication. The relative size of the data markers indicates weight of sample size from each study. DCB: drug-coated balloon; DES: drug-eluting stent; BMS: bare-metal stent.

Data on a composite of MACEs were available in all 485 patients (100%). The funnel plot of the primary outcome was roughly symmetrical (Supplementary Figure 3). The definition of MACEs differed slightly among the trials (Table 1). There was no difference in the incidence of MACEs with DCB compared with the control group (random effects: 5.42% vs. 9.39%; RR 0.61 [0.31 to 1.17]; *P*=0.14; Figure 2(a)), with no significant heterogeneity among the studies (*I*^2^ = 0). The incidence of MACEs was similar when DCB compared with DES (RR 0.67 [0.31 to 1.45]; *P*=0.31; Figure 2(c)), but DCB were associated with a lower incidence of MACEs compared with BMS (RR 0.35 [0.13 to 0.98]; *P*=0.05; Figure 2(c)).

### 3.3. Secondary Endpoints

Compared with the control group, DCB was associated with no significant difference in the incidence of all-cause mortality (2.92% vs. 4.90%; RR 0.61 [0.24 to 1.51]; *P*=0.29; Figure 2(e)). The incidence was not statistically significantly different when comparing DCB either with DES (RR 0.73 [0.24 to 2.20]; *P*=0.58; Supplementary Figure 2A) or with BMS (RR 0.47 [0.14 to 1.60]; *P*=0.23; Supplementary Figure 2A). The risk of cardiovascular mortality was also not significant different between DCB and control groups (2.08% vs. 3.27%; RR 0.66[0.22 to 2.00]; *P*=0.46; Figure 2(f)). Nor was the difference statistically significant when comparing DCB either with DES (RR 0.77 [0.23 to 2.57]; *P*=0.67; Supplementary Figure 2B) or with BMS (RR 0.71 [0.15 to 3.38]; *P*=0.66; Supplementary Figure 2B).

The risk of MI was significantly reduced for DCB as compared with the control group (0% vs. 3.27%; RR 0.16 [0.03 to 0.91]; *P*=0.04; Figure 2(b)). The difference was numerically lower for DCB as compared with DES (RR 0.18 [0.03 to 1.05]; *P*=0.06; Figure 2(d)) and BMS (RR 0.14 [0.01 to 2.90]; *P*=0.20*P* = 0.20; Figure 2(d)), but this difference was not statistically significant. The risk of ST was low in both DCB and control groups, and only 4 ST events were found in DES. There was no significant difference when comparing DCB with the control group (0% vs. 1.63%; RR 0.29 [0.05 to 1.73]; *P*=0.17; Figure 2(g)) or DES (RR 0.24 [0.04 to 1.46]; *P*=0.12; Supplementary Figure 2C).

There was no difference in the incidence of TLR with DCB compared with the control group (2.08% vs. 2.86%; RR 0.81 [0.26 to 2.57]; *P*=0.72; Figure 2(h)). The incidence of TLR was similar when DCB compared with DES (RR 0.87 [0.27 to 2.83]; *P*=0.82; Supplementary Figure 2D) and BMS (RR 0.71 [0.05 to 11.06]; *P*=0.80; Supplementary Figure 2D), respectively.

### 3.4. Angiographic Outcomes

**Figure 3:**
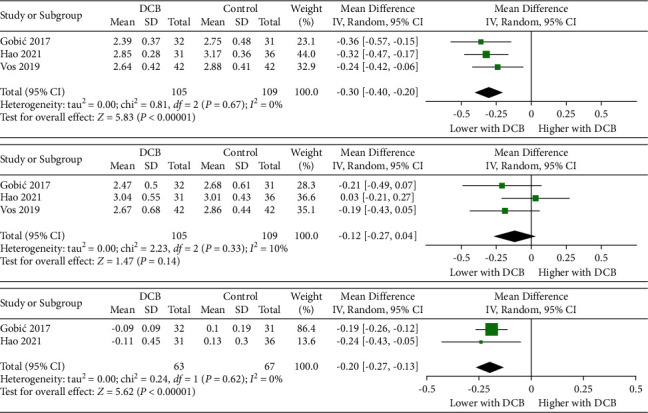
Summary plots for the angiographic outcomes. Mean difference of minimum lumen diameter after the index procedure (a), minimum lumen diameter at the follow-up angiograph (b), and late lumen loss (c). The relative size of the data markers indicates weight of sample size from each study. DCB: drug-coated balloon; DES: drug-eluting stent; BMS: bare-metal stent.

Routine angiographic follow-up was performed ranging from 6 to 12 months, and one trial did not show angiographic outcomes [18]. When compared with the control group (only second-generation DES was included), DCB was associate with lower MLD_postindexprocedure_ (2.63 mm vs. 2.94 mm; WMD −0.30 [−0.40 to −0.20]; *P* < 0.00001; Figure 3(a)) but similar MLD_follow-up angiograph_ (2.72 mm vs. 2.86 mm; WMD −0.12 [−0.27 to 0.04]; *P*=0.14; Figure 3(b)). The LLL was significantly lower for DCB as compared with the control group (−0.10 mm vs. 0.12 mm; WMD −0.20 [−0.27 to −0.13]; *P* < 0.00001; Figure 3(c)).

## 4. Discussion

In this metaanalysis of 4 randomized trials including 485 patients with acute myocardial infarction undergoing PCI, we documented that DCB was associated with no difference in the incidence of MACEs compared with the control on routine clinical follow-up. This effect was consistent when compared DCB with DES. DCB was associated with lower risk of MACEs compared with BMS. DCB was also associated with no difference in the incidence of all-cause mortality, cardiovascular mortality, stent thrombosis, and TLR. Importantly, the incidence of MI was lower with DCB, but this effect was not consistent when comparedDCB with DES or BMS, respectively. DCB was associated with lower MLD_postindexprocedure_ but similar MLD_follow-upangiograph_, thus lower LLL compared with control (all patients were treated with DES) on routine angiographic follow-up. However, these findings were based on a small number of trials, with a small number of events, and should therefore be viewed only as hypothesis-generating. Overall, our findings strongly suggest the value of DCB-only strategy as an attractive “leave nothing behind” strategy for selected patients with AMI provided a satisfactory result is obtained after lesion predilation.

Receiving second-generation DES is the most common option for the treatment of patients with AMI and is generally considered the optimal strategy [2]. In some special cases, such as high-bleeding risk, BMS is still used to minimize the duration of antiplatelet therapy. Nicola and his colleagues conducted the first study of a DCB-only strategy in the setting of PPCI [23]. This study showed good one-year clinical results with only 5 MACEs occurred, but additional stenting was performed in half of the patients. Recently, the DEBUT trial showed that PCI with DCB was superior to BMS in patients at high-bleeding risk [24]. In this trial, 46% patients had acute coronary syndrome (ACS) and only one patient occurred MACEs in the DCB group, which means that the DCB-only strategy may be safe and effective in ACS patients. Our metaanalysis demonstrated that DCB was associated with similar clinical outcomes in patients with AMI compared with second-generation DES and favorable clinical outcomes when compared with BMS. In addition, although paclitaxel is the common drug for balloon coating, there is increasing clinical research evidence that sirolimus-coated balloon is clinically feasible and safe. Recently, the SIRPAC study [25], an indirect comparison of paclitaxel-coated and sirolimus-coated balloons for PCI, showed that there was no significant difference in clinical endpoints (including MACE, TLR, MI, death, and bleeding) at 12-monthfollow-up. Of note, nearly half of the patients enrolled in this study were diagnosed with ACS, suggesting that the sirolimus-coated balloon is also safe and feasible in patients with ACS.

Compared with BMS, the use of DES is associated with accelerated progression and an increased prevalence of in-stent neoatherosclerosis, which may lead to an increased rate of very late stent thrombosis [26]. Although the emergence of second-generation DES subsequently reduced the incidence of late ST, it permanently prevents full recovery of vascular structure and function with accordant risk of very late stent failure [27]. The DCB-only strategy offers a potential advantage in the context of high thrombus load and inflammation. Local antiproliferative drug delivery by DCB without the need for metal struts at the time of peak inflammatory state, as in STEMI, has many potential benefits in endothelial function preservation, such as reduced risk of thrombosis due to less malapposition and homogeneous administration of the drug [12]. However, our metaanalysis only showed a trend of decreasing risk of ST, but this reduction was not significant due to the low incidence of events.

The DCB-only strategy shows another potential advantage in overcoming intimal hyperplasia [28, 29], which is clinically manifested as significant vessel enlargement and plaque regression [30]. Our metaanalysis showed that compared with DES, DCB has significantly lower LLL but similar MLD at follow-up angiography, suggesting that although DCB has a worse immediate effect, it might show better results during follow-up.

One pervious metaanalysis has compared the clinical and angiographic outcomes in patients with AMI treated with DCB vs. stenting [31]. However, that metaanalysis included an observational study, which were prone to ascertainment and selection biases. Besides, the included studies of stenting in that metaanalysis combined the use of first-generation and second-generation DES, which precluded a comparison between DCB and second-generation DES. The present metaanalysis only included randomized trials and has provided a comprehensive overview of the clinical and angiographic outcomes of DCB vs. current-generation DES.

The present metaanalysis has the following limitation that must be considered. First, despite a comprehensive literature search, the number of studies including in this metaanalysis is still small. Second, although low heterogeneity was observed in most analyses, there were some differences in the including studies, such as the duration of follow-up. Therefore, we adopted the random-effects statistical models for analysis in this study. Third, the studies included in this analysis were insufficient, especially in terms of a subgroup analysis. Thus, the findings could only be considered hypothesis-generating. What is more, publication bias and sensitivity analysis could not be performed. Fourth, some outcome measures, such as LLL, were not normally distributed and were reported as medians and quartiles and therefore could not be included in the analysis. Fifth, the lack of patient-level data impeded a careful assessment of the patient and lesion characteristics that would benefit most from DCB. Finally, BMS was no longer used in routine practice except for patients with high-bleeding risk [24], but this metaanalysis still intakes a clinical trial that included BMS because very few RCTs met the requirements.

## 5. Conclusion

In this metaanalysis, DCB was associated with similar incidence of MACEs, all-cause mortality, cardiovascular mortality, stent thrombosis, TLR, and lower incidence of MI compared with control. On routine angiographic follow-up, DCB showed similar MLD_follow-upangiograph_ and lower MLD_postindexprocedure_ and LLL compared with second-generation DES. DCB might be a feasible interventional strategy in the treatment of patients with AMI. Future large-volume, well-designed RCTs with extensive follow-up are awaited for evaluating the role of the DCB in this setting.

## Figures and Tables

**Table 1:** Characteristics of randomized controlled trials included in the metaanalysis.

Author/acronym	Years	Lesion characteristic	No. of patents			Follow-up period (month)		Primary end-point	Definition of MACEs
			DCB	DES	BMS	Angiographic	Clinical		
Gobic et al. [16]	2017	STEMI	38	37	NA	6	6	MACEs	Death, TLR, ST
REVELATION [17]	2019	NSTEMI	104	51	69	NA	9	TLF	All-cause death, MI, TLR, stroke, PCI at other vessels
PEPCAD NSTEMI [18]	2020	STEMI	60	60	NA	9	9	FFR	Death, MI, TLR
Hao et al. [19]	2021	STEMI	38	42	NA	12	12	LLL	Death, MI, TLR

DCB: drug-coated balloon; DES: drug-eluting stent; BMS: bare-metal stent; MACEs: major adverse cardiac events; STEMI: ST-segment elevation myocardial infarction; TLR: target lesion revascularization; ST: stent thrombosis; NSTEMI: non-ST elevation myocardial infarction; TLF: target lesion failure; MI: myocardial infarction; PCI: percutaneous coronary intervention; FFR: fractional flow reserve; LLL: late lumen loss.

## Data Availability

The datasets used or analyzed during the current study are available in article/Supplementary materials, and more data are available from the corresponding author on reasonable request.

## Supplementary Materials

Supplementary Materials
*Supplementary Table 1*. PRISMA checklist; *Supplementary Table 2.* Search strategy; *SupplementaryTtable 3*. Baseline patients and trial characteristics; *Supplementary Figure 1.* Risk of bias of the individual studies; *Supplementary Figure 2*. Summary plots for the subgroup analysis; *Supplementary Figure 3.* Funnel plots.
