# Supplementary material for: Drug-Coated Balloons for Acute Myocardial Infarction: A Metaanalysis of Randomized Clinical Trials
Source: J Interv Cardiol. 2022 Dec 27;2022:4018771. doi: 10.1155/2022/4018771 (PMC9810407; doi:10.1155/2022/4018771)

Supplementary Material

Supplementary Table 1 - Checklist of items to include when reporting a systematic review or meta-analysis

| Section/topic | # | Checklist item | Reported on page # |
| --- | --- | --- | --- |
| **TITLE** | | | |
| Title | 1 | Identify the report as a systematic review, meta-analysis, or both. | 1 |
| **ABSTRACT** | | | |
| Structured summary | 2 | Provide a structured summary including, as applicable: background; objectives; data sources; study eligibility criteria, participants, and interventions; study appraisal and synthesis methods; results; limitations; conclusions and implications of key findings; systematic review registration number. | 1 |
| **INTRODUCTION** | | | |
| Rationale | 3 | Describe the rationale for the review in the context of what is already known. | 1 |
| Objectives | 4 | Provide an explicit statement of questions being addressed with reference to participants, interventions, comparisons, outcomes, and study design (PICOS). | 1 |
| **METHODS** | | | |
| Protocol and registration | 5 | Indicate if a review protocol exists, if and where it can be accessed (e.g., Web address), and, if available, provide registration information including registration number. | 2 |
| Eligibility criteria | 6 | Specify study characteristics (e.g., PICOS, length of follow-up) and report characteristics (e.g., years considered, language, publication status) used as criteria for eligibility, giving rationale. | 2 |
| Information sources | 7 | Describe all information sources (e.g., databases with dates of coverage, contact with study authors to identify additional studies) in the search and date last searched. | 2 |
| Search | 8 | Present full electronic search strategy for at least one database, including any limits used, such that it could be repeated. | 2 |
| Study selection | 9 | State the process for selecting studies (i.e., screening, eligibility, included in systematic review, and, if applicable, included in the meta-analysis). | 2 |
| Data collection process | 10 | Describe method of data extraction from reports (e.g., piloted forms, independently, in duplicate) and any processes for obtaining and confirming data from investigators. | 2 |
| Data items | 11 | List and define all variables for which data were sought (e.g., PICOS, funding sources) and any assumptions and simplifications made. | 2 |
| Risk of bias in individual studies | 12 | Describe methods used for assessing risk of bias of individual studies (including specification of whether this was done at the study or outcome level), and how this information is to be used in any data synthesis. | 3 |
| Summary measures | 13 | State the principal summary measures (e.g., risk ratio, difference in means). | 3 |
| Synthesis of results | 14 | Describe the methods of handling data and combining results of studies, if done, including measures of consistency (e.g., I^2^) for each meta-analysis. | 3 |
| Risk of bias across studies | 15 | Specify any assessment of risk of bias that may affect the cumulative evidence (e.g., publication bias, selective reporting within studies). | 3 |
| Additional analyses | 16 | Describe methods of additional analyses (e.g., sensitivity or subgroup analyses, meta-regression), if done, indicating which were pre-specified. | 3 |
| RESULTS | | | |
| Study selection | 17 | Give numbers of studies screened, assessed for eligibility, and included in the review, with reasons for exclusions at each stage, ideally with a flow diagram. | 3 |
| Study characteristics | 18 | For each study, present characteristics for which data were extracted (e.g., study size, PICOS, follow-up period) and provide the citations. | 3 |
| Risk of bias within studies | 19 | Present data on risk of bias of each study and, if available, any outcome-level assessment (see Item 12). | Table S3 |
| Results of individual studies | 20 | For all outcomes considered (benefits or harms), present, for each study: (a) simple summary data for each intervention group and (b) effect estimates and confidence intervals, ideally with a forest plot. | 3 |
| Synthesis of results | 21 | Present results of each meta-analysis done, including confidence intervals and measures of consistency. | 3-4 |
| Risk of bias across studies | 22 | Present results of any assessment of risk of bias across studies (see Item 15). | 3-4 |
| Additional analysis | 23 | Give results of additional analyses, if done (e.g., sensitivity or subgroup analyses, meta-regression) (see Item 16). | NA |
| DISCUSSION | | | |
| Summary of evidence | 24 | Summarize the main findings including the strength of evidence for each main outcome; consider their relevance to key groups (e.g., health care providers, users, and policy makers). | 4 |
| Limitations | 25 | Discuss limitations at study and outcome level (e.g., risk of bias), and at review level (e.g., incomplete retrieval of identified research, reporting bias). | 5 |
| Conclusions | 26 | Provide a general interpretation of the results in the context of other evidence, and implications for future research. | 5 |
| FUNDING | | | |
| Funding | 27 | Describe sources of funding for the systematic review and other support (e.g., supply of data); role of funders for the systematic review. | 5 |

Supplementary Table 2. Search strategy

| PubMed | Search strategies | Results |
| --- | --- | --- |
| #1 | coated balloon[Title/Abstract] | 1036 |
| #2 | eluting balloon[Title/Abstract] | 507 |
| #3 | #1 OR #2 | 1419 |
| #4 | Myocardial Infarction[MeSH Terms] | 181550 |
| #5 | primary percutaneous coronary intervention[Title/Abstract] | 6435 |
| #6 | acute myocardial infarction[Title/Abstract] | 61379 |
| #7 | #4 OR #5 OR #6 | 198303 |
| #8 | #3 AND #7 | 97 |
| #9 | ((randomized controlled trial[Publication Type]) OR randomized controlled trial[MeSH Terms])OR controlled clinical trial[Publication Type] | 771543 |
| #10 | (((Animal Experimentation[MeSH Terms]) OR Animals,Laboratory[MeSH Terms]) OR Models, Animal[MeSH Terms]) OR rodentia[MeSH Terms] | 3490158 |
| #11 | #8 AND #9 NOT #10 | 29 |
| Embase | Search strategies | Results |
| #1 | ‘coated balloon’:ab,ti | 1602 |
| #2 | ‘eluting balloon’:ab,ti | 932 |
| #3 | #1 OR #2 | 2437 |
| #4 | ‘myocardial infarction’/exp | 418127 |
| #5 | ‘Primary percutaneous coronary intervention’:ab,ti | 11517 |
| #6 | ‘acute myocardial infarction’:ab,ti | 87059 |
| #7 | #4 OR #5 OR #6 | 426150 |
| #8 | #3 AND #7 | 587 |
| #9 | 'randomized controlled trial' OR 'randomized controlled trials as topic'/exp OR 'random*':ti,ab | 1887620 |
| #10 | 'animals, laboratory'/exp OR 'animal experiment'/exp OR 'animal model'/exp OR 'rodent'/exp | 4855701 |
| #11 | #8 AND #9 NOT #10 | 184 |
| Cochrane Library | Search strategies | Results |
| #1 | coated balloon:ti,ab,kw | 841 |
| #2 | eluting balloon:ti,ab,kw | 1383 |
| #3 | #1 OR #2 | 1831 |
| #4 | [mh “myocardial infarction”] | 11373 |
| #5 | primary percutaneous coronary intervention:ti,ab,kw | 6130 |
| #6 | acute myocardial infarction:ti,ab,kw | 16190 |
| #7 | #4 OR #5 OR #6 | 23707 |
| #8 | #3 AND #7 | 567 |
| #9 | randomized OR clinical trials:ti,ab,kw | 1499927 |
| #10 | rat OR rats OR mouse OR mice:ti,ab,kw OR [mh animals] | 616179 |
| #11 | #8 AND #9 NOT #10 | 183 |
| Web of Science | Search strategies | results |
| #1 | TS=coated balloon | 9132 |
| #2 | TS=eluting balloon | 11631 |
| #3 | #1 OR #2 | 18120 |
| #4 | TS=myocardial infarction | 525667 |
| #5 | TS=primary percutaneous coronary intervention | 23705 |
| #6 | TS=acute myocardial infarction | 212839 |
| #7 | #4 OR #5 OR #6 | 529161 |
| #8 | #3 AND #7 | 4650 |
| #9 | TS=(clinical trial* OR controlled trial* OR random* trial* OR random*stud*) | 1983100 |
| #10 | #8 AND #9 limit Clinical Trial (Document Types) | 627 |
| ClinicalTrials.gov | | |
| 13 Studies found for: coated balloon OR eluting balloon \| acute myocardial infarction | | |

Supplementary Table 3. Baseline patient and trial characteristic

| Author/acronym | Single/  multicenter | Country | Trial registration | Age, years | Men, % | Diabetes mellitus, % | Hypertension, % | Body mass index, kg/m2 |
| --- | --- | --- | --- | --- | --- | --- | --- | --- |
| Gobić D, et al. | Single center | Croatia | NR | 57/54 | 71/73 | 5/11 | 32/35 | NR |
| REVELATION | Single center | Netherlands | NCT02219802 | 57/57 | 87/87 | 13/7 | 30/32 | 27/27 |
| PEPCAD NSTEMI | Multicenter | Germany | NCT01489449 | 66/67 | 66/68 | 27/36 | 79/88 | 29/28 |
| Yellow D, et al. | Single center | China | NR | 59/56 | 75/82 | NR | NR | 26/25 |

Data are reported as drug-coated balloon/control. NR, not reported

Supplementary Figure 1. Risk of bias of the individual studies by Cochrane risk assessment tool.


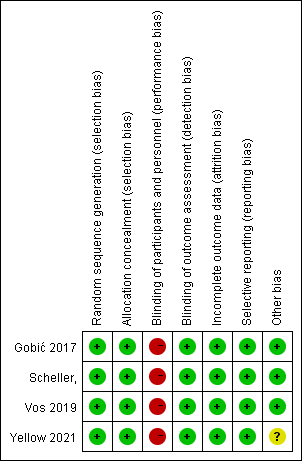


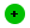
=Low risk of bias;
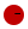
=Risk of bias;
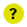
=Unclear

Supplementary Figure 2. Summary plots for the Subgroup analysis for clinical endpoint.


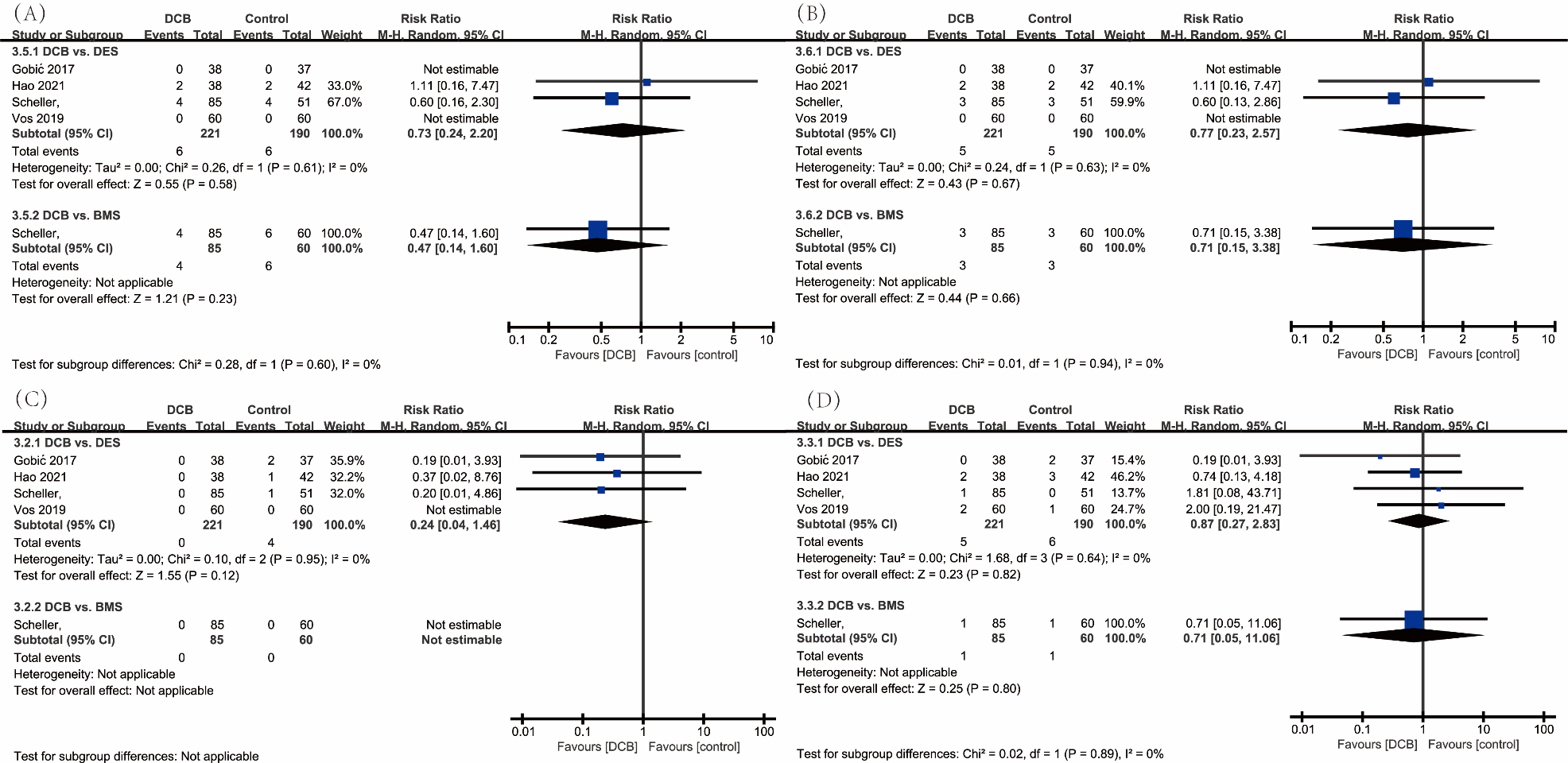


Subgroup analysis for all-cause death (A), cardiovascular mortality (B), stent thrombosis (C), target lesion revascularization (D) according to indication. The relative size of the data markers indicates weight of sample size from each study. DCB, drug-coated balloon; DES, drug-eluting stent; BMS, bare-metal stent.

Supplementary Figure 3. Funnel Plot for Primary Endpoint.


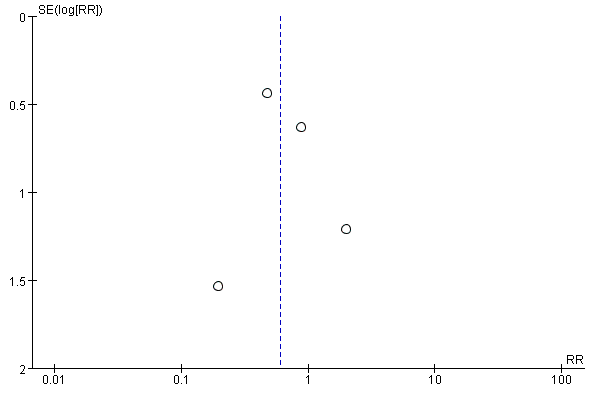

Supplement: Supplementary Materials — Supplementary Table 1. PRISMA checklist; Supplementary Table 2. Search strategy; SupplementaryTtable 3. Baseline patients and trial characteristics; Supplementary Figure 1. Risk of bias of the individual studies; Supplementary Figure 2. Summary plots for the subgroup analysis; Supplementary Figure 3. Funnel plots. [file 4018771.f1.docx]
